# Cyotoxicity of Etidronate as an Endodontic Chelator Against Periodontal Ligament Fibroblast

**DOI:** 10.1111/aej.70029

**Published:** 2025-10-29

**Authors:** Bruno Vila Nova de Almeida, Patrícia de Almeida Rodrigues, Geovanni Pereira Mitre, Maria Sueli da Silva Kataoka, João de Jesus Viana Pinheiro, Talita Tartari, Oscar Faciola Pessoa

**Affiliations:** ^1^ Department of Endodontic Dental School, Federal University of Para Belem Pará Brazil; ^2^ Department of Pathology Dental School, Federal University of Para Belem Pará Brazil; ^3^ Department of Restorative Dentistry Piracicaba School of Dentistry, State University of Campinas Piracicaba São Paulo Brazil

**Keywords:** chelating agents, cytotoxicity, EDTA, etidronate, periodontal ligament fibroblasts

## Abstract

Etidronic acid (HEBP) causes fewer alterations in dentine and can be used mixed with sodium hypochlorite during root canal preparation. This study investigated the cytotoxicity effects of the HEDP and EDTA on human periodontal ligament fibroblast cells. Primary culture was established from human periodontal ligament tissue of one donor and was characterised by immunofluorescence and real‐time quantitative polymerase chain reaction. The half‐maximal inhibitory concentration (IC_50_) was tested: 9% HEBP, 18% HEBP, 24% HEBP, 15% EDTA, 17% EDTA, 24% EDTA, and control. Exposure times were 1, 4, and 24 h. The Kruskal‐Wallis test, followed by Dunn's post hoc, assessed multiple comparisons between the groups (*α* = 0.05). All experimental groups showed a lower IC_50_ effect after 24 h, except 9% HEBP. EDTA cytotoxicity depends on the contact time, regardless of the concentration. In conclusion, 9% HEBP is a biocompatible alternative to EDTA as an endodontic chelator, particularly for protocols requiring longer exposure times.

## 1 Introduction

Root canal irrigation is a critical component of endodontic treatment, contributing to the removal of debris, cleaning of anatomically complex areas, prevention and elimination of the smear layer, and disinfection of the root canal system [1, 2]. The most common irrigation protocols in endodontics use sodium hypochlorite solution (NaOCl) due to its antimicrobial activity and ability to dissolve organic matter, but it has cytotoxic potential and the incapacity to remove the smear layer reinforces the need to use chelating agents [3].

Ethylenediaminetetraacetic acid (EDTA) is the most commonly used chelating solution that increases the release of bioactive molecules in the dentine matrix [4], but it can compromise dentine properties and cause complete loss of available chlorine of NaOCl if mixed with this irrigant [5]. It also promotes greater sealer bonding strength to the walls of the root canal [6], presents a relevant antimicrobial activity when combined with surface‐active agents [7], despite the cytotoxic potential, although not genotoxic [8].

HEBP, also known as etidronate or etidronic acid, is a less aggressive chelant to dentine than EDTA [9], causing a higher release of growth factors from dentine disks and cylinders [10], demonstrating biocompatibility in Chinese hamster lung fibroblast (V79) cells at low concentrations, and is also non‐genotoxic [11]. HEBP does not affect the antimicrobial properties or tissue dissolution ability of NaOCl when freshly mixed [12], preserving its chelating capacity specially in third root apical [13]. However, 9% and 18% HEBP did not outperform EDTA in terms of smear layer and debris removal or antimicrobial action, despite not causing dentine erosion or altering dentine properties in continuous chelation protocols [14].

However, a potential complication during root canal preparation is the extrusion of organic and inorganic debris, microorganisms, and irrigants into the periradicular tissues [15, 16]. This extrusion is often associated with adverse outcomes, including interappointment flare‐ups, postoperative discomfort, and prolonged healing [17, 18]. The intensity of postoperative sensitivity is also related to the physical and physiological factors of the patients and the level of dental contamination [19].

The fibroblasts are the main cells of the periodontal ligament, responsible for the structure of biological connective tissue, maintaining the homeostasis, turnover, repair, and tissue regeneration [20]. Fibroblasts constitute the predominant cell type in the periodontal ligament, potentially differentiating in vivo into osteoblasts or cementoblasts [21]. Given the cellular heterogeneity, it is crucial to characterise fibroblast cultures established from human tissue. Despite their low immunogenicity, fibroblasts can produce more inflammatory factors than leukocytes [22].

Primary fibroblast culture preserves phenotypic aspects of the origin tissue, cell function, growth characteristics, cell markers, signalling, and genetic integrity, although variations in vivo metabolic activity that are unreproducible in vitro [23]. Primary cultures do not undergo the modifications induced in permanent cells, can represent a response closer to the target site, and are a source of stem cells for personalised regenerative therapies [24].

The inflammatory response caused by the contact of endodontic irrigants extrusion with the fibroblasts of the periodontal ligament can promote a dysregulation of the cell cycle, which can lead to growth inhibition, cytotoxicity, or apoptosis. It is therefore important to know if the chelating agent is more or less cytotoxic to the fibroblasts. Therefore, the objective of this study was to investigate the cytotoxicity of HEBP and EDTA solutions at various concentrations and exposure times in human periodontal ligament fibroblasts. The null hypothesis tested is that there is no difference between the cytotoxic effects of both chelating agents on the cells.

## 2 Materials and Methods

The research was carried out in accordance with ethical and legal principles approved by the Committee of Ethics in Human Research (CAAE 0121.0.073.000‐11). The patient received comprehensive information regarding the advantages, potential risks, and alternative treatment options before being enrolled in the research. Additionally, it was emphasized that opting out of participation would not affect their treatment in any way. One patient (male, 27 years old) provided informed consent. The study followed the guidelines set forth in the World Medical Association Declaration of Helsinki and received approval from the Institutional Ethics Committee. The Preferred Reporting Items for Laboratory Studies in Endodontology (PRILE) 2021 Guidelines were used to write the manuscript [25].

### 2.1 Cell Culture

Cell cultures were established from one impacted third molar extracted for orthodontic reasons, according to the previous description [21]. Briefly, human periodontal ligament tissue was removed from the middle third of the root surface with a curette, washed with PBS buffer (phosphate‐buffered saline) (Sigma Aldrich, St. Louis, Missouri, USA), fragmented, and then cultured in 25 cm^2^ tissue flasks using Dulbecco's Modified Eagle's Medium F12 (DMEM F12—Sigma‐Aldrich, St. Louis, MO, USA), pH 7.2, supplemented with 10% fetal bovine serum (FBS—Laborclin, Pinhais, Parana, Brazil), 100 IU/mL penicillin, 100 μg/mL streptomycin, and 5 μg/mL amphotericin B. Cultures were grown at 37°C in a 5% CO_2_ humidified incubator.

The medium was changed twice a week until the cell layers reached confluence, and monitoring was carried out using an inverted phase contrast microscope (Axiovert 40 C—Zeiss, Oberkochen, Germany) with an attached camera (AxioCam MRc—Zeiss). Subcultures were performed using the standard trypsinization protocol. Establishment of a primary culture allowed the use of five secondary subcultures, which ensured a sufficient amount of cells for experiments. A total of 2 × 10^4^ exponential cells were seeded to reach 80% confluence. For all experiments, cells between the fourth and sixth passages were employed.

### 2.2 Indirect Immunofluorescence

Because the cell population of periodontal ligament tissue is heterogeneous, cells of forty passages were characterised using indirect immunofluorescence. The mouse anti‐vimentin (VIM) monoclonal antibody (1:100), mouse anti‐cytokeratin AE1/AE3 (CKAE1/AE3) monoclonal (1:100), mouse anti‐fibronectin (FN) monoclonal antibody (1:100), and mouse anti‐sclerotin (SOST) monoclonal antibody (1:100) were used as primary antibodies. For the detection of the primary antibody, samples were incubated in a solution containing the Alexa Fluor 488‐conjugated secondary antibodies (Invitrogen, Waltham, MA, EUA) for 1 h in a humidified, dark chamber at room temperature. For improved visualisation of the cytoskeleton, Alexa Fluor 568 Phalloidin (Life Technologies, Carlsbad, CA, USA) was used. Nuclei were counterstained with Hoechst 33258 dye, conjugated to the ProLong Gold antifade reagent (Invitrogen). Cells were examined and photographed under a fluorescence microscope (Axio Scope.A1, Zeiss) to evaluate the expression.

### 2.3 Real‐Time Quantitative Polymerase Chain Reaction (RT‐qPCR)

To investigate and characterise the cellular phenotype of periodontal ligament cells, reverse transcription followed by real‐time quantitative polymerase chain reaction (RT‐qPCR) was performed. Total RNA was extracted from the periodontal ligament fibroblast (PLF) cell line cultured in 25 cm^2^ flasks at approximately 90% confluence, using TRIzol reagent (Invitrogen), following the manufacturer's protocol.

RNA quantification was carried out using the Qubit Fluorometer (Invitrogen) together with the Quant‐iT RNA Assay kit (Invitrogen). Subsequently, reverse transcription of 1 μg of total RNA per sample was performed using the GoTaq 2‐Step RT‐qPCR System kit (Promega, Madison, WI, USA) to obtain cDNA. qPCR reactions were conducted on an Applied Biosystems 7500 Real‐Time PCR System (Thermo Fisher Scientific) using the GoTaq 2‐Step RT‐qPCR System (Promega). Each reaction was prepared with 2.5 ng of cDNA, following the kit's instructions.

Primers used targeted the following genes: alkaline phosphatase (Forward (F): GTA CAA CAC CAA TGC CCA GG; Reverse (R): CAG ATT TCC CAG CGT CCT CCT TG), osteopontin (F: GTT TGA GAA GGT GTG CAG CA; R: TGT ATT TGC AAG GCC CGA TG), bone morphogenetic protein 2 (BMP2) (F: GGT TTT CCG AGA ACA GAT GCA; R: AGT CTG GTC ACG GGG AAT TT), periostin (F: GAA GGA ATG AAA GGC TGC CC; R: CAA GCC TCA TTA CTC GGT GC), and calcium binding protein a4 (F: GTC AGA ACT AAA GGA GCT GCT G; R: CGT TGT CCC TGT TGC TGT C).

Cycling conditions were as follows: initial denaturation at 95°C for 2 min, followed by 40 amplification cycles consisting of 30 s at 95°C, 40 s at 60°C, and 15 s at 95°C. At the end of the reaction, a melting curve analysis was performed to confirm the specificity of the amplified products (15 s at 95°C, 1 min at 60°C, 15 s at 95°C, and 15 s at 60°C). Data visualisation was carried out using the equipment software.

For data analysis, cycle threshold (Ct) values were obtained for all target genes. A Ct cutoff value of less than 35 cycles (Ct < 35) was established to consider gene expression as positive. Ct values above this threshold were interpreted as negative or indicating very low/no expression of the target gene in the samples analyzed.

### 2.4 Cytotoxicity Assay

Fibroblast cell cultures were seeded in 96‐well plates (TPP) at a concentration of 3 × 10^3^ cells/well in 200 μL of DMEM F12 supplemented with 10% FBS. In the exponential phase, the cells were exposed to 9% HEBP, 18% HEBP, 24% HEBP, 15% EDTA, 17% EDTA, and 24% EDTA at 1/200 in DMEM F12. DMEM F12 was used in the positive control group (CT). The exposure times to the solutions were 1, 4, and 24 h. For the verification of cell viability, the MTT assay (Sigma‐Aldrich, St. Louis, MO, USA) was used. After exposure, the solutions were replaced by MTT at a concentration of 5 mg/mL in PBS, which remained in the incubator for 4 h and was replaced with isopropyl alcohol (Merck KGaA, Darmstadt, Germany) for the solubilisation of formazan crystals (Bio‐Rad; iMarkTM, UK). The reading was performed using Microplate Manager software (Bio‐Rad—UK) and adopting dual filters: 595 and 655 nm reference filters. The experiments were performed in triplicate.

### 2.5 Statistical Analysis

Descriptive statistics were used to determine the percentage of cell viability. The absorbance data was determined by the percentage of cell viability in relation to the control group (100%). Considering the ability of different time and concentration intervals to reduce cell viability to less than 50% was categorised as cytotoxic (IC_50_). For group comparisons, the Kruskal‐Wallis test was utilised, followed by Dunn's post hoc test for multiple comparisons between the test groups. Statistical analyses were performed using the GraphPad Prism 10.1.2 software, considering a significance level of 5%.

## 3 Results

**FIGURE 1:**
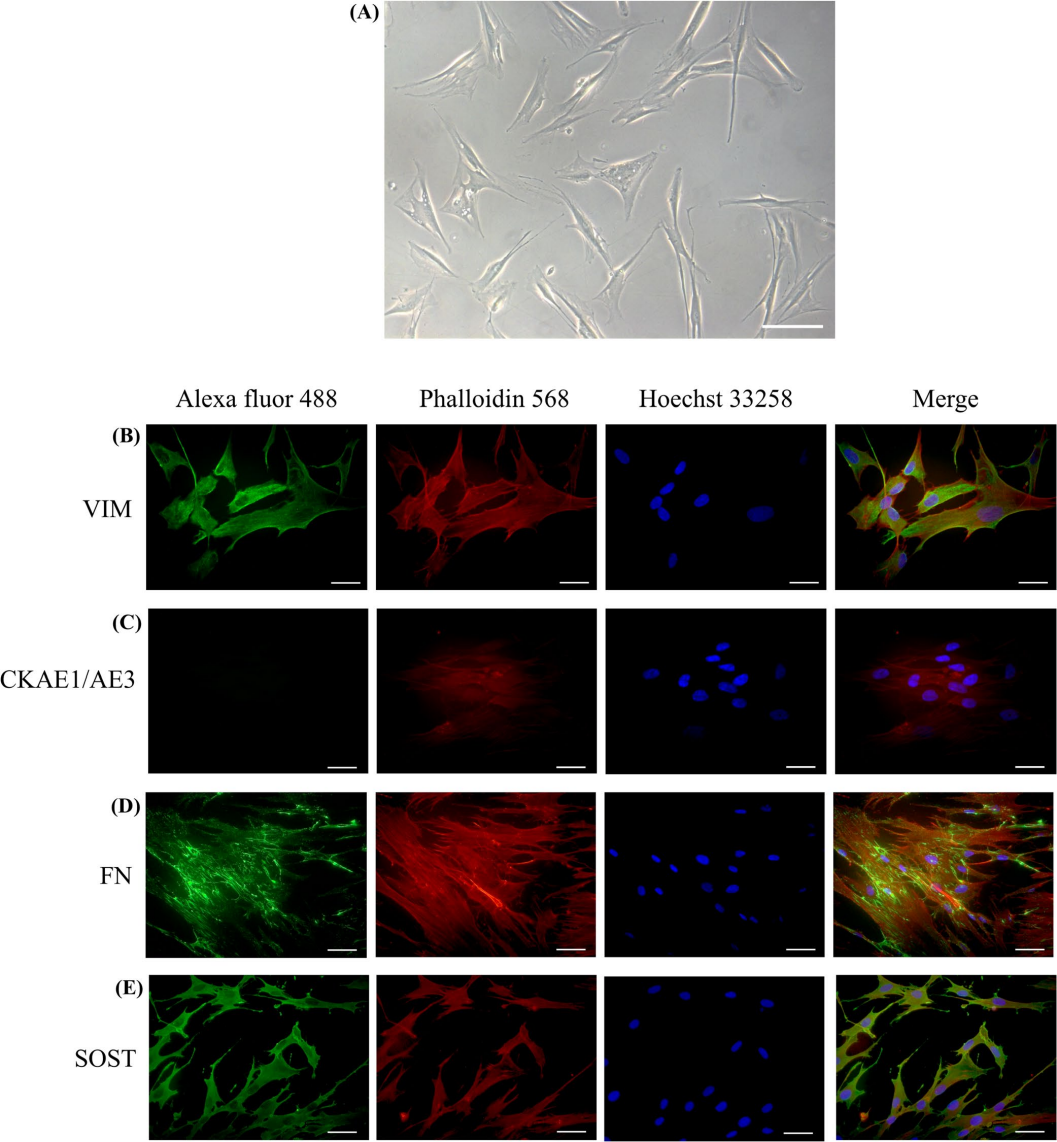
Morphological characterization of periodontal ligament fibroblasts by phase‐contrast and immunofluorescence microscopy. (A) Human periodontal ligament fibroblasts in non‐confluent monolayer at the fourth passage, observed under phase‐contrast microscopy (100 μm scale bar), displaying fusiform morphology and slender cytoplasmic extensions. Immunofluorescence showed vimentin with diffuse, filamentous cytoplasmic staining (B); absent or low AE1/AE3 cytokeratin labeling (C); fibronectin with filamentous distribution in the pericellular matrix (D); and sclerostin with punctate or diffuse cytoplasmic staining (E), consistent with periodontal origin (100 μm scale bar).

Under phase‐contrast microscopy, human periodontal ligament fibroblasts at the fourth passage exhibited typical fusiform morphology, with elongated cytoplasm and slender cytoplasmic projections (Figure 1A).

In the immunofluorescence results, vimentin labeling exhibited a diffuse, filamentous cytoplasmic pattern distributed throughout the cytoskeleton, confirming the mesenchymal phenotype of the cells (Figure 1B). In contrast, AE1/AE3 cytokeratin expression was absent or minimal, reinforcing the lack of epithelial features in the culture (Figure 1C). Fibronectin exhibited a filamentous distribution in the extracellular matrix, particularly in the pericellular region, forming networks associated with focal adhesion sites, consistent with its structural and cell‐anchoring role (Figure 1D). Sclerostin labeling, in turn, showed a punctate or diffuse cytoplasmic pattern, in agreement with periodontal ligament fibroblasts, in which this protein may be involved in tissue remodeling regulation and local osteogenic signaling (Figure 1E).

Quantitative PCR analysis was performed to characterise the gene expression profile of periodontal ligament fibroblasts. All target genes tested, including Alkaline Phosphatase, Osteopontin, BMP2, Periostin, and Calcium Binding Protein, were positively detected with Ct values below the 35‐cycle cutoff (Table 1). These findings confirm the presence of the tested genes and support the identity and origin of the cultured cells as fibroblasts derived from periodontal ligament tissue.

**TABLE 1:** Ct values and detection status of target genes in periodontal ligament fibroblast cell culture.

Gene	Mean Ct	Detection (yes/no)
Alkaline phosphatase	31.542	Yes
Osteopontin	25.213	Yes
BMP1	30.547	Yes
Periostin	26.521	Yes
Calcium binding protein	22.186	Yes

**FIGURE 2:**
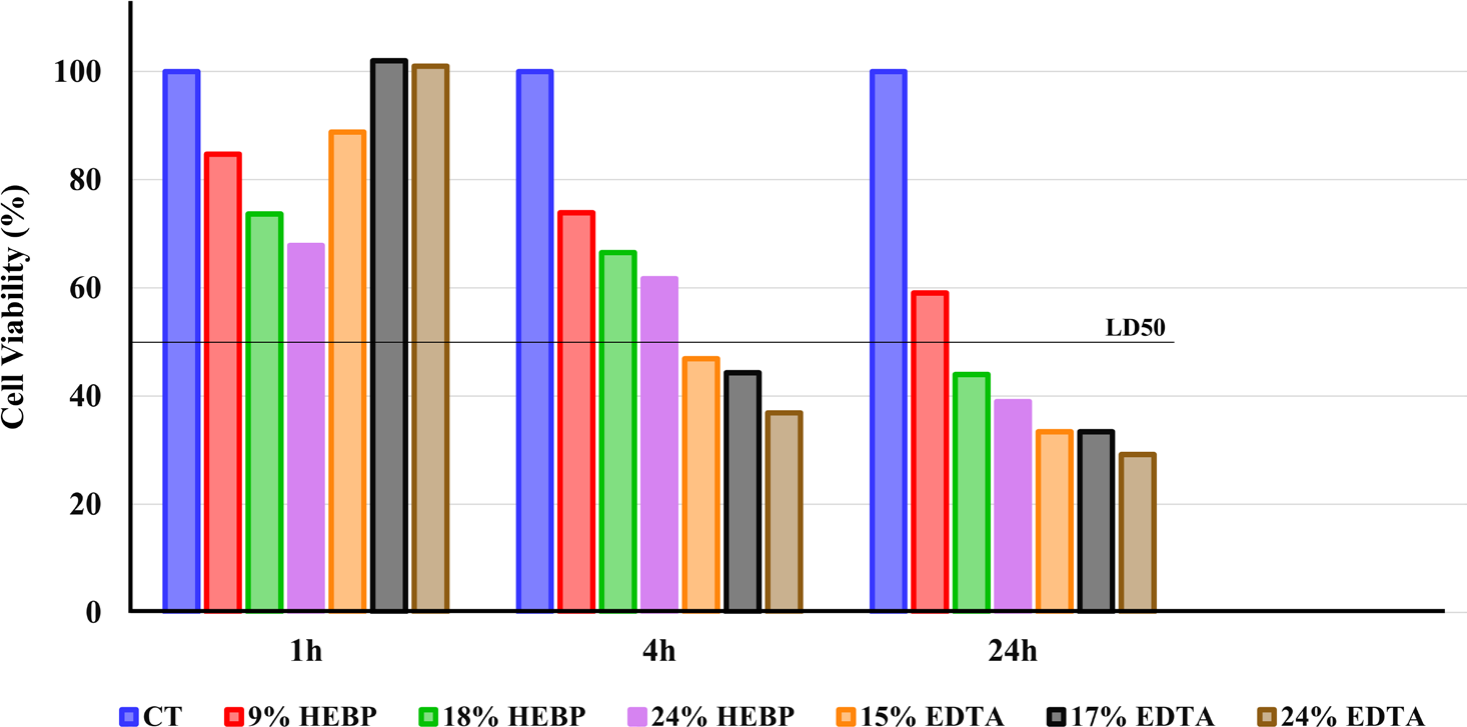
Cell viability after treatment with—CT (without treatment); 9% HEBP; 18% HEBP; 24% HEBP; 15% EDTA; 17% EDTA; 24% EDTA in 1 h, 4 h, and 24 h. Percentage of cell viability in reference to control (100%). Concentrations that reduced cell viability below 50% were considered cytotoxic.

Figure 2 shows cell viability in percentage from the control as a parameter. 9% HEBP was the only solution that did not reach IC_50_ at any time point, while 18% HEBP and 24% HEBP exceeded IC_50_ at 24 h. For EDTA, there was no reduction at 1 h; however, at 4 h a significant reduction was observed, with values below IC_50_ in all concentrations. When comparing the time points, all EDTA concentrations showed a difference between 1 × 4 h and 1 × 24 h, whereas none of the HEBP concentrations caused a significant reduction in cell viability. Also comparing the groups all concentrations reduced cell viability in relation to the control, with 9% HEBP showing a reduction only at 24 h; 18% HEBP and 24% HEBP from 1 h onwards; and all EDTA concentrations at 4 h. All EDTA concentrations exhibited higher cytotoxicity compared to 9% HEBP after 4 h. 18% HEBP and 24% HEBP reduced cell viability more than 17% EDTA and 24% EDTA at 1 h, and 24% EDTA caused a greater reduction than all HEBP concentrations at 4 h. There was no difference between the concentrations of HEBP and EDTA at the same time point, and 18% HEBP and 24% HEBP were similar to 15% EDTA (Table 2).

**TABLE 2:** Median and Interquartile Range (Med ± IQR) of fibroblast viability following 1, 4, and 24 h exposure to HEBP and EDTA treatments.

Treatment	Periods of exposure tested		
	1 h Med ± IQR	4 h Med ± IQR	24 h Med ± IQR
Control	0.22 ± 0.06^A,a^	0.23 ± 0.05^A,a^	0.24 ± 0.08^A,a^
9% HEBP	0.19 ± 0.05^AB,a^	0.17 ± 0.07^A,a^	0.14 ± 0.08^B,a^
18% HEBP	0.16 ± 0.04^B,a^	0.15 ± 0.07^bc,a^	0.11 ± 0.05^bc,a^
24% HEBP	0.15 ± 0.04^B,a^	0.14 ± 0.06^bc,a^	0.09 ± 0.08^bc,a^
15% EDTA	0.20 ± 0.06^AB,a^	0.10 ± 0.01^CD,b^	0.08 ± 0.07^bc,b^
17% EDTA	0.22 ± 0.08^A,a^	0.10 ± 0.02^CD,b^	0.08 ± 0.05^bc,b^
24% EDTA	0.23 ± 0.08^A,a^	0.08 ± 0.02^D,b^	0.07 ± 0.05^C,b^

*Note:* Kruskal‐Wallis test with Dunn's post hoc, *p* < 0.05: Different lowercase letters in the rows denote significant intragroup differences across the periods of exposure tested. Kruskal‐Wallis test with Dunn's post hoc, *p* < 0.001: Different uppercase letters in the columns indicate significant intergroup differences for the same period of exposure.

## 4 Discussion

Chelating solutions play a prominent role in cleaning and disinfecting the root canal system [26]. Ideally, the contact of root canal irrigants with periapical tissues should not cause aggression, since cytotoxicity alters cellular metabolism, induces vascular changes, activates inflammatory cells, reduces cell proliferation, produces chemical mediators, alters macrophage function, leads to cell death through necrosis or apoptosis, and degrades dentine collagen [27].

Among the concentrations and exposure times investigated in this study, the 9% HEBP solution demonstrated the most favorable outcomes in terms of biocompatibility. Therefore, the null hypothesis that there is no difference between the cytotoxic effects of both chelating agents on the cells must be rejected. The findings of our study align with a study that indicated concentrations ranging from 1 to 1000 μM showed no cytotoxic effects at 48 and 72 h [28]. Similarly, etidronic acid was classified as a non‐toxic solution for various cell types at concentrations of 1, 10, and 100 μM after 72 h [29]. Additionally, 9% HEBP combined with 2.5% NaOCl reaches a pH of 10.5 by achieving equilibrium between hypochlorite and hypochlorous acid, thereby reducing its mixture cytotoxicity [30].

HEBP decreased cell viability faster than EDTA; however, the high polarity of HEBP reduces cellular penetration [11], making this reduction non‐significant regardless of the concentration when comparing across the exposure periods tested. The chelating capacity of HEBP depends on the concentration [31], and the increase from 9% to 18% does not significantly affect the amide III/phosphate ratio or the dentine carbonate/phosphate ratio [32]. However, it is important to note that 18% HEBP and 24% HEBP concentrations exceed IC_50_ within 24 h.

The cytotoxicity and genotoxicity of a combination of 9% etidronic acid (Dual Rinse HEDP, Medcem, Weinfelden, Switzerland) with a 2.5% NaOCl solution were attributed to the presence of free available chlorine derived from the NaOCl in the mixture. When chlorine was lost, minimal adverse effects were detected, reinforcing that etidronic acid exhibits lower cytotoxicity even when mixed with NaOCl [11]. However, endodontic treatment of mature permanent teeth with pulpal necrosis in 42 patients revealed that the mixture of 1.5% NaOCl and 9% etidronic acid caused more postoperative pain than the sequential use of 1.5% NaOCl with 17% EDTA or 10% citric acid, despite the results being statistically comparable to the use of analgesics [33]. The significant postoperative pain observed may be attributed to the greater amount of debris extruded apically by the mixture of 18% etidronate and 5% NaOCl [34, 35].

A randomised clinical trial involving 60 patients aged between 18 and 65 years compared those treated with 9% etidronate prepared in 2.5% NaOCl to those treated with a pure 2.5% NaOCl solution. The trial found that etidronic acid did not compromise the antimicrobial capacity of NaOCl, nor did it induce inflammatory effects on periapical tissues, as evidenced by the absence of postoperative pain 24 h after the procedure and without an increase in neutrophil activity [36]. These findings underscore the clinical significance of its biocompatible potential.

The concentration of 17% EDTA has limited smear layer removal in the apical third [37], which can lead to microleakage in the filling material and treatment failure in endodontics. In this study, the 24% EDTA was tested because it has been demonstrated that 24% EDTA does not interfere with periodontal tissue repair in conventional treatment [38], or in pre‐ and post‐operative root conditioning [39], and it improved the colonisation of human periodontal ligament fibroblasts on the root surface [40]. The effects of the 24% concentration of these chelants on dentine and in irrigation protocols with NaOCl are unknown; besides, it has been shown that irrigation with NaOCl after the chelants reverses the collagen in the mineralised dentine [32].

The extrusion of EDTA can alter physiological processes due to the chelation of various metal ions that influence enzymatic reactions [41], such as calcium, which is important in blood coagulation, neuromuscular contraction, and vascular permeability [42]. There is a divergence concerning the initial cytotoxicity caused by EDTA, with studies showing intense cell death from the outset [27, 43]. Regarding EDTA tested over the shortest time, no significant reduction in cell viability was observed at any concentration tested, as the cells remained viable for a shorter period of EDTA exposure [44]. A 5‐min incubation in primary dental pulp stem cell cultures showed that 17% EDTA significantly increased cell proliferation, while 9% HEDP promoted a greater release of the growth factor TGF‐β, and both were effective in cell migration [45].

In this research, 4 h EDTA showed a drastic reduction in cell viability, reinforcing the time‐dependent toxic potential of EDTA, but with no difference between the tested concentrations. This agrees with studies of 17% EDTA using fibroblast cultures, which detected a cytotoxic effect between 3 and 6 h [46, 47], as well as maintaining significant cytotoxicity at 24 h [48]. These results reinforce that the contact time of the chelant determines the cellular response, apparently more than concentration.

Utilising primary cell cultures derived from the specific site of interest facilitates the assessment of unique reactions to a particular material, with a high potential for specificity that closely mimics the response of target cells in dental tissues [49]. While cytotoxicity studies provide valuable insights into the cellular response to treatment, it is important to acknowledge that further testing is needed to fully assess the biocompatibility of these chelating agents. Specifically, future studies should compare the cellular responses in primary cultures obtained from different patients, as individual variability may influence the outcomes. Additionally, the complex interplay of systemic factors and the limitations of in vitro models make it challenging to directly extrapolate laboratory findings to clinical scenarios. Therefore, comprehensive in vivo studies and broader clinical evaluations are essential to establish the true biocompatibility of these.

In addition, exploring the impact of different proportions of the chelating agent in relation to the sodium hypochlorite solution on cell viability, biofilm decontamination capability, and cellular attachment to the tooth surface is essential. There is also a need to expand in vitro investigations to assess the effect on pulp revascularization and root perforation sealing.

## 5 Conclusion

Considering their biocompatibility, the result reinforces that 9% HEBP emerges as an alternative chelating agent to EDTA with no discernible long‐term cytotoxic effects, even with direct contact with the cells if extruded to periapical tissues, whereas the cytotoxicity of EDTA is related to the contact time.

## Author Contributions

**Bruno Vila Nova de Almeida:** conceptualisation; methodology; investigation; writing – original draft; visualisation. **Patrícia de Almeida Rodrigues:** conceptualisation; methodology; formal analysis; investigation; writing – original draft; visualisation; supervision. **Geovanni Pereira Mitre:** conceptualisation; methodology; investigation; resources; writing – original draft; visualisation. **Maria Sueli da Silva Kataoka:** conceptualisation; investigation; writing – review and editing. **João de Jesus Viana Pinheiro:** conceptualisation; investigation; resources; writing – review and editing. **Talita Tartari:** conceptualisation; writing – review and editing. **Oscar Faciola Pessoa:** conceptualisation; methodology; resources; writing – review and editing; supervision. All authors have contributed significantly; all authors are in agreement with the manuscript.

## Disclosure

The authors have nothing to report.

## Ethics Statement

The research was carried out in accordance with ethical and legal principles approved by the Committee of Ethics in Human Research (CAAE 0121.0.073.000‐11).

## Conflicts of Interest

The authors declare no conflicts of interest.

## Data Availability

The data that support the findings of this study are available from the corresponding author upon reasonable request.
